# Long-Term Neurodevelopmental Outcomes After Forceps, Vacuum, and Second-Stage Cesarean Delivery

**DOI:** 10.1001/jamanetworkopen.2025.56637

**Published:** 2026-01-30

**Authors:** Maya Rajasingham, Sarka Lisonkova, Neda Razaz, Giulia M. Muraca

**Affiliations:** 1 Department of Obstetrics and Gynecology, Faculty of Health Sciences, McMaster University, Hamilton, Ontario, Canada; 2 Department of Health Research Methods, Evidence, and Impact, Faculty of Health Sciences, McMaster University, Hamilton, Ontario, Canada; 3 Department of Obstetrics and Gynecology, Faculty of Medicine, University of British Columbia, Vancouver, British Columbia, Canada; 4 Clinical Epidemiology Unit, Department of Medicine, Solna, Karolinska Institutet, Eugeniahemmet, Stockholm, Sweden

## Abstract

**Importance:**

Prior studies have compared neurodevelopmental outcomes across delivery modes that are not interchangeable (eg, operative vs spontaneous vaginal delivery [SVD]), limiting the clinical utility of their conclusions.

**Objective:**

To examine the association between mode of operative delivery in the second stage of labor and attention-deficit/hyperactivity disorder (ADHD), autism spectrum disorder (ASD), and intellectual disability (ID) in children.

**Design, Setting, and Participants:**

This population-based, retrospective cohort study used data from health administrative databases from British Columbia, Canada, from April 1, 2000, to December 31, 2019. The population included 504 380 nonanomalous, singleton, full-term infants who survived and did not emigrate during their first year of life. Neurodevelopmental outcomes were collected through March 31, 2022, and analyses were performed from June 2024 to August 2025.

**Exposure:**

Delivery mode during the second stage of labor, categorized as SVD, forceps, vacuum, sequential instrument, or second-stage cesarean delivery (SSCD).

**Main Outcomes and Measures:**

The main outcome was ADHD, ASD, and ID from 1 year to a maximum of 22 years of age. Cox proportional hazards regression models were used to assess the association between mode of operative delivery (SVDs excluded) and each neurodevelopmental outcome, adjusting for maternal and clinical characteristics.

**Results:**

Among 504 380 children (253 256 males [50.2%] and 44 202 [8.8%] with a maternal history of neurodevelopmental or psychiatric disorders), 407 792 (80.9%) were delivered by SVD, 46 493 (9.2%) by vacuum, 23 140 (4.6%) by forceps, 3009 (0.6%) by sequential instrument, and 23 946 (4.7%) by SSCD. The overall rate of ADHD was 6.6 (95% CI, 6.5-6.7) per 1000 person-years; ASD, 1.8 (95% CI, 1.7-1.8) per 1000 person-years; and ID, 0.3 (95% CI, 0.2-0.3) per 1000 person-years. Compared with SSCD, children who underwent sequential instrument delivery had a 13% higher ADHD rate (7.9 [95% CI, 7.1-8.8] vs 6.6 [95% CI, 6.3-6.9] per 1000 person-years; adjusted hazard ratio [AHR], 1.13 [95% CI, 1.00-1.28]) and those who underwent vacuum delivery had a 53% higher ID rate (0.3 [95% CI, 0.3-0.4] vs 0.2 [95% CI, 0.1-0.3] per 1000 person-years; AHR, 1.53 [95% CI, 1.12-2.10]). There was no significant association with ASD and mode of delivery.

**Conclusions and Relevance:**

The findings of this cohort study suggest that children who underwent operative vaginal delivery and SSCD had comparable neurodevelopmental outcomes, except among those who underwent sequential instrument delivery and vacuum delivery, which were associated with ADHD and ID, respectively. Further research is warranted to develop a better understanding of delivery mode as a potentially modifiable risk factor for neurodevelopmental disorders in childhood.

## Key Points

**Question:**

Is there an association between mode of delivery in the second stage of labor and attention-deficit/hyperactivity disorder (ADHD), autism spectrum disorder (ASD), and intellectual disability (ID)?

**Findings:**

This cohort study of 504 380 children found higher rates of ADHD among children who underwent sequential instrument delivery compared with second-stage cesarean delivery, higher rates of ID among those who underwent vacuum delivery, and similar rates of ASD between mode of delivery groups.

**Meaning:**

The findings of this study suggest that operative vaginal delivery and second-stage cesarean delivery have comparable neurodevelopmental outcomes among offspring, except among those who underwent sequential instrument and vacuum delivery, which were associated with ADHD and ID, respectively.

## Teaser

This cohort study examines the association between mode of operative delivery in the second stage of labor and attention-deficit/hyperactivity disorder, autism spectrum disorder, and intellectual disability among children born in British Columbia, Canada.

## Introduction

Some of the most prevalent neurodevelopmental disorders among children include attention-deficit/hyperactivity disorder (ADHD) (5%-7%),^1,2^ autism spectrum disorder (ASD) (1%-2%),^3,4^ and less frequently, intellectual disability (ID) (0.4%-1%).^5,6^ Although the causes of ADHD, ASD, and ID remain unclear, there is agreement that they manifest due to genetic and nongenetic factors.^2,4,6^ Reviews have highlighted the need for more longitudinal research based on large patient populations to assist in identifying modifiable risk factors of these disorders.^6,7^ One factor may include mode of delivery, which has been associated with many prenatal and postnatal factors related to neurodevelopmental outcomes, including oxidative stress and birth injuries.^2,4,6,8^

Overall, research investigating general behavioral and neurodevelopmental concerns, such as emotional, conduct, and/or prosocial behaviors, have found comparable rates between mode of delivery groups.^9,10^ However, research focusing on specific neurodevelopmental outcomes have highlighted children born by a cesarean or operative vaginal delivery (using forceps or vacuum) to have a 6% to 10% higher risk of ADHD, 6% to 20% higher risk of ASD, and 8% to 26% higher risk of ID compared with children born by spontaneous vaginal delivery.^11,12,13,14^ Although the association between mode of delivery and neurodevelopmental outcomes has been studied, most research relies on broad comparisons between cesarean and vaginal deliveries.^11,12,15,16,17^ A systematic review of studies comparing neurodevelopmental and psychiatric outcomes among offspring born via cesarean vs vaginal delivery found 61 studies on this topic.^17^ Their findings suggested that cesarean deliveries are associated with a 33% increased risk of ASD and 17% increased risk of ADHD; however, the review stressed that this body of evidence is compromised by confounding by indication and the conceptualization of cesarean and vaginal delivery as 2 homogeneous groups.

The binary framing of cesarean vs vaginal delivery is problematic, as these groups are not inherently exchangeable. Vaginal deliveries include both spontaneous and operative vaginal births, while cesarean deliveries vary by timing (prelabor, first stage of labor, second stage of labor) and context (elective, emergency).^18^ Cesarean deliveries are also performed in response to specific clinical indications (such as fetal distress, labor arrest, or maternal complications) that are themselves associated with neurodevelopmental risk.^2,4,6,18^ As a result, any observed association between cesarean delivery and neurodevelopmental outcomes may reflect confounding by indication, rather than a causal effect of the delivery mode itself.

Rather than comparing cesarean delivery with vaginal delivery overall, the focus of studies comparing neurodevelopmental outcomes should be on specific, clinically relevant decision points, where the options are more likely to be comparable—for example, second-stage cesarean delivery (SSCD) vs operative vaginal delivery among individuals who require an intervention in the second stage of labor to facilitate birth. These comparisons better reflect the actual choices clinicians and patients face and are less prone to confounding because they compare people in similar clinical contexts. Moreover, as the rate of SSCD increases,^19^ there is a need to understand the comparative risks between SSCD and operative vaginal delivery.

We aimed to evaluate the association between mode of delivery in the second stage of labor and ADHD, ASD, and ID. We hypothesized that the risk of each neurodevelopmental outcome would vary by mode of delivery.

## Methods

### Study Design and Data Sources

We conducted a population-based, retrospective cohort study of births in British Columbia, Canada, using data from the British Columbia Perinatal Data Registry (which captures more than 99% of births in the province) from April 1, 2000, to December 31, 2019.^20^ Birth records were linked with 6 datasets that included information on (1) hospitalizations, (2) outpatient physician visits, (3) prescription medications, (4) health care coverage information, (5) additional birth-related data, and (6) death records (eMethods in Supplement 1). Databases were linked using unique encoded identifiers and accessed through Population Data BC.^21^ We followed the Strengthening the Reporting of Observational Studies in Epidemiology (STROBE) reporting guideline.^22^ This study received ethics approval from the University of British Columbia Children’s and Women’s Research Ethics Board, which waived the need for informed consent owing to the use of deidentified data.

### Study Population

We identified singleton infants between 37 and 42 weeks’ gestation who were born during the second stage of labor. Exclusions were made for preterm or postterm births, deliveries that occurred prior to full cervical dilation (prelabor or first stage of labor), and breech deliveries. In addition, we excluded children who had received a diagnosis of a congenital anomaly within 1 year of birth (*International Classification of Diseases, Ninth Revision* [*ICD-9*], codes 740-749 and 750-759; *International Statistical Classification of Diseases and Related Health Problems, Tenth Revision* [*ICD-10*], codes Q00-Q99).

### Exposure

Our exposure was mode of delivery during the second stage of labor: (1) spontaneous vaginal delivery, (2) vacuum delivery, (3) forceps delivery, (4) sequential instrument delivery (failed vacuum followed by forceps), and (5) SSCD. Mode of delivery information was obtained from the British Columbia Perinatal Database Registry, for which validation studies have demonstrated a high degree of validity.^23^

### Outcome

The primary outcomes were a diagnosis of ADHD, ASD, and ID at any time after a child’s first birthday until the end of the study period (March 31, 2022). Diagnoses of ADHD, ASD, and ID reflect conditions recorded in a patient’s medical record by physicians during hospitalizations and outpatient visits. The diagnoses are collated using *ICD-9* or *ICD-10* codes by trained medical record abstractors using standardized forms and coding rules. Validated algorithms to identify each disorder were applied using *ICD-9* or *ICD-10* codes obtained from hospitalization and physician outpatient records, as well as prescription medications using Anatomical Therapeutic Chemical codes (eTable 1 in Supplement 1).^1,24^

### Covariates

We selected potential confounders a priori based on previous literature and consultation with clinical experts.^17,25,26^ These confounders included maternal characteristics: age (≤19, 20-24, 30-34, 35-39, and ≥40 years vs 25-29 years), parity (nulliparous vs parous), prepregnancy body mass index (BMI; calculated as weight in kilograms divided by height in meters squared: underweight, <18.5; overweight, 25.0-29.9; and obese, ≥30; vs normal, 18.5-24.9), smoking status (smoked during pregnancy or quit prior to pregnancy vs none), history of maternal neurodevelopmental or psychiatric disorders (yes or no), diabetes (preexisting and gestational; yes or no), and gestational hypertension (yes or no). Infant characteristics included sex (female vs male), infant birth weight of 4000 g or more, fetal distress, and fetal dystocia. The data sources of each covariate and relevant diagnosis codes are provided in eTable 1 in Supplement 1.

### Statistical Analysis

Statistical analysis was performed from June 2024 to August 2025. We calculated crude rates of ADHD, ASD, and ID per 1000 person-years by mode of delivery, where estimates for the spontaneous vaginal delivery group were calculated to provide insight into outcome rates among births that occurred without an intervention. Direct comparisons between spontaneous vaginal delivery and operative vaginal delivery or SSCD were not made, as confounding by indication would have biased such analyses. However, we did construct cumulative incidence curves among forceps delivery, vacuum delivery, sequential instrument delivery, and SSCD groups to estimate the probability of ADHD, ASD, and ID over time. In addition, we estimated adjusted hazard ratios (AHRs) and 95% CIs of each neurodevelopmental disorder using Cox proportional hazards regression models with robust standard errors to account for intracluster correlations at the maternal level. For each disorder, children were followed up from 1 year of age until diagnosis of the outcome, death, emigration, or study completion, whichever occurred first (eFigure in Supplement 1). Children who died, emigrated, or had a diagnosis of ADHD, ASD, or ID before 1 year of age (start of follow-up) were removed from the analysis of each outcome. For example, children who had received a diagnosis of ADHD prior to 1 year of age were removed from ADHD analyses, but not from analyses of ASD or ID. A complete-case approach was performed because all variables had less than 1.5% missing values, except for BMI (26.3% missing values), for which missing was included as a category.

We undertook 3 sensitivity analyses. First, we evaluated potential bias due to missing data on BMI by using multiple imputation and combining the results of 10 imputation cycles (PROC MIANALYZE in SAS). Second, we removed smoking status from our statistical models due to the potential misclassification of nonsmokers. Finally, we explored mode of delivery as successful or failed instrument use following an intention-to-treat framework. For this analysis, we restricted our cohort to births between 2002 and 2019 given the consistent use of *ICD-10* codes over this period required to create these successful or failed categories. Analyses were performed in SAS, version 9.4 (SAS Institute Inc), and RStudio, version 4.3.1 (R Project for Statistical Computing), with the extension packages episensr, mice, and survival, with statistical significance set at a 2-sided α of .05.

## Results

**Figure 1.**
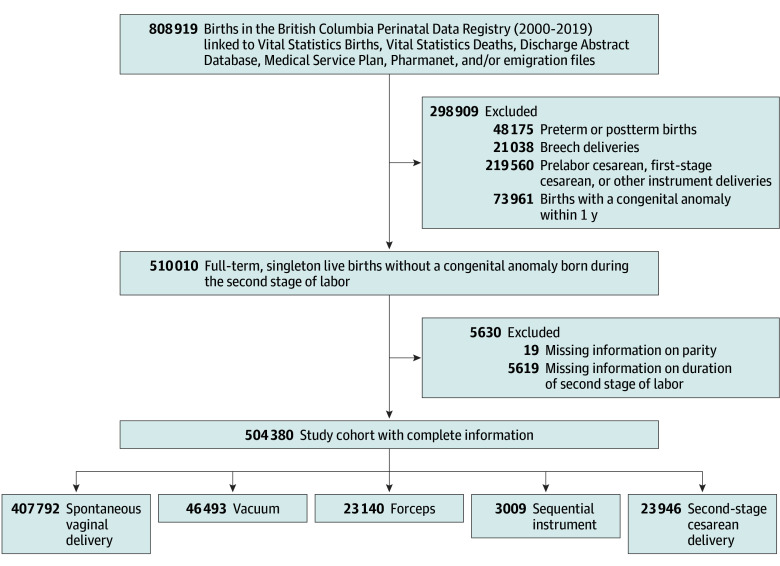
Derivation of Study Cohort

Overall, 834 669 singleton children were born in British Columbia between April 1, 2000, and December 31, 2019. We excluded 25 750 children (3.1%) with unknown exposure or outcome information and 298 909 children (37.0%; Figure 1) with either a preterm or postterm delivery, a breech delivery, a cesarean delivery that occurred prelabor or during the first stage of labor, or a congenital anomaly diagnosis prior to 1 year of age. We also excluded observations with missing information on parity and/or duration of the second stage of labor (5630 [1.1%]). Thus, 504 380 children (253 256 males [50.2%] and 251 124 females [49.8%]) were included in our study, of whom 407 792 (80.9%) were born by a spontaneous vaginal delivery, 46 493 (9.2%) by a vacuum delivery, 23 140 (4.6%) by a forceps delivery, 3009 (0.6%) by a sequential instrument delivery, and 23 946 (4.7%) by an SSCD (Figure 1 and Table 1). The number of children who remained in our statistical models after excluding spontaneous vaginal deliveries and children who died, emigrated, or had an outcome diagnosis before their first birthday, and/or had missing covariate information was 96 520 for ADHD, 96 531 for ASD, and 96 528 for ID.

**Table 1.** Demographic and Clinical Characteristics of Full-Term, Nonanomalous Children Born During the Second Stage of Labor, British Columbia, Canada, 2000 to 2019

Characteristic	Total births, No. (%) (N = 504 380)	Delivery type, No. (%)				
		Spontaneous vaginal delivery (n = 407 792)^a^	Vacuum delivery (n = 46 493)	Forceps delivery (n = 23 140)	Sequential instrument delivery (n = 3009)	Second-stage cesarean delivery (n = 23 946)
**Maternal characteristics**						
Age, y						
≤19	16 336 (3.2)	13 719 (3.4)	1535 (3.3)	502 (2.2)	97 (3.2)	483 (2.0)
20-24	69 509 (13.8)	57 743 (14.2)	6515 (14.0)	2328 (10.1)	449 (14.9)	2474 (10.3)
25-29	144 921 (28.7)	116 640 (28.6)	14 119 (30.4)	6531 (28.2)	942 (31.3)	6689 (27.9)
30-34	172 006 (34.1)	137 727 (33.8)	15 634 (33.6)	8707 (37.6)	968 (32.2)	8970 (37.5)
35-39	85 552 (17.0)	69 107 (16.9)	7248 (15.6)	4288 (18.5)	461 (15.3)	4448 (18.6)
≥40	16 056 (3.2)	12 856 (3.2)	1442 (3.1)	784 (3.4)	92 (3.1)	882 (3.7)
Parity						
Nulliparous	232 168 (46.0)	156 941 (38.5)	32 688 (70.3)	19 785 (85.5)	2501 (83.1)	20 253 (84.6)
Parous	272 212 (54.0)	250 851 (61.5)	13 805 (29.7)	3355 (14.5)	508 (16.9)	3693 (15.4)
Prepregnancy BMI						
<18.5	24 680 (4.9)	19 041 (4.7)	3029 (6.5)	1418 (6.1)	216 (7.2)	976 (4.1)
18.5-24.9	233 612 (46.3)	185 565 (45.5)	22 686 (48.8)	12 381 (53.5)	1532 (50.9)	11 448 (47.8)
25.0-29.9	74 047 (14.7)	60 152 (14.8)	6068 (13.1)	3246 (14.0)	367 (12.2)	4214 (17.6)
≥30	40 073 (7.9)	33 505 (8.2)	2721 (5.9)	1422 (6.1)	154 (5.1)	2271 (9.5)
Missing	131 968 (26.2)	109 529 (26.9)	11 989 (25.8)	4673 (20.2)	740 (24.6)	5037 (21.0)
History of ADHD, ASD, or ID	1915 (0.4)	1621 (0.4)	152 (0.3)	58 (0.3)	7 (0.2)	77 (0.3)
History of neurodevelopmental or psychiatric disorders	44 202 (8.8)	36 708 (9.0)	3590 (7.7)	1758 (7.6)	248 (8.2)	1898 (7.9)
Preexisting diabetes	1231 (0.2)	880 (0.2)	124 (0.3)	89 (0.4)	12 (0.4)	126 (0.5)
Smoking status						
During pregnancy	43 988 (8.5)	36 485 (8.9)	3593 (7.7)	1265 (5.5)	208 (6.9)	1437 (6.0)
Quit prior to pregnancy	36 494 (7.2)	29 392 (7.2)	3195 (6.9)	1699 (7.3)	194 (6.4)	2014 (8.4)
None	424 898 (84.3)	341 915 (83.8)	39 705 (85.4)	20 176 (87.2)	2607 (86.6)	20 495 (85.6)
Gestational diabetes	40 878 (8.1)	31 633 (7.8)	4076 (8.8)	2298 (9.9)	231 (7.7)	2640 (11.0)
Gestational hypertension	19 531 (3.9)	14 387 (3.5)	2145 (4.6)	1283 (5.5)	148 (4.9)	1568 (6.5)
Duration of second stage of labor, h						
<1	326 879 (64.8)	303 566 (74.4)	17 874 (38.4)	3717 (16.1)	655 (21.8)	1067 (4.5)
1-1.9	85 835 (17.0)	67 352 (16.5)	11 846 (25.5)	3755 (16.2)	646 (21.5)	2236 (9.3)
2-2.9	44 762 (8.9)	24 763 (6.1)	9426 (20.3)	5158 (22.3)	795 (26.4)	4620 (19.3)
3-3.9	24 309 (4.8)	7923 (1.9)	4550 (9.8)	4788 (20.7)	514 (17.1)	6534 (27.3)
≥4	22 595 (4.5)	4188 (1.0)	2797 (6.0)	5722 (24.7)	399 (13.3)	9489 (39.6)
**Child characteristics**						
Birth year						
2000-2004	120 003 (23.8)	97 567 (23.9)	11 327 (24.4)	6139 (26.5)	598 (19.9)	4372 (18.3)
2005-2009	129 705 (25.7)	105 053 (25.8)	12 598 (27.1)	5202 (22.5)	1228 (40.8)	5624 (23.5)
2010-2014	130 663 (25.9)	105 546 (25.9)	12 329 (26.5)	5550 (24.0)	799 (26.6)	6439 (26.9)
2015-2019	124 009 (24.6)	99 626 (24.4)	10 239 (22.0)	6249 (27.0)	384 (12.8)	7511 (31.4)
Sex						
Female	251 124 (49.8)	207 576 (50.9)	20 885 (44.9)	10 662 (46.1)	1265 (42.0)	10 736 (44.8)
Male	253 256 (50.2)	200 216 (49.1)	25 608 (55.1)	12 478 (53.9)	1744 (58.0)	13 210 (55.2)
Gestational age, wk						
37-38	115 606 (22.9)	97 453 (23.9)	9212 (19.8)	4397 (19.0)	480 (16.0)	4064 (17.0)
39-42	388 774 (77.1)	310 339 (76.1)	37 281 (80.2)	18 743 (81.0)	2529 (84.0)	19 882 (80.3)
Birth weight ≥4000 g	63 710 (12.6)	50 882 (12.5)	5045 (10.9)	2631 (11.4)	437 (14.5)	4715 (19.7)
Fetal distress	82 427 (16.3)	45 668 (11.2)	18 806 (40.4)	9132 (39.5)	1078 (35.8)	7743 (32.3)
Dystocia	84 580 (16.8)	43 582 (10.7)	14 071 (30.3)	11 063 (47.8)	1439 (47.8)	14 425 (60.2)

Abbreviations: ADHD, attention-deficit/hyperactivity disorder; ASD, autism spectrum disorder; BMI, body mass index (calculated as weight in kilograms divided by height in meters squared); ID, intellectual disability.

^a^ Spontaneous vaginal delivery data are reported only for descriptive purposes and are not intended for comparison with operative delivery.

Births after an SSCD tended to have a higher proportion of birthing individuals with a maternal age of 35 years or older, BMI of 30 or higher, gestational diabetes, and gestational hypertension (Table 1). Duration of labor varied by mode of delivery, with longer times among SSCD and forceps delivery. Moreover, individuals who gave birth after a vacuum delivery were more likely to smoke during pregnancy and have a higher parity.

Over the study period, the overall rate of ADHD was 6.6 (95% CI, 6.5-6.7) per 1000 person-years (7693 cases; median follow-up time, 11.6 years [IQR, 7.2-16.8 years]), the overall rate of ASD was 1.8 (95% CI, 1.7-1.8) per 1000 person-years (2131 cases; median follow-up time, 12.5 years [IQR, 7.6-17.4 years]), and the overall rate of ID was 0.3 (95% CI, 0.2-0.3) per 1000 person-years (323 cases; median follow-up time, 12.6 years [IQR, 7.8-17.5 years]). The rates of ADHD, ASD, and ID among children born after a spontaneous vaginal delivery are described in Table 2.

**Table 2.** Neurodevelopmental Disorders Among Full-Term, Nonanomalous Children Born During the Second Stage of Labor, British Columbia, Canada, 2000 to 2019

Mode of delivery	Neurodevelopmental disorder								
	ADHD			ASD			ID		
	No. of births	No. of events	Rate per 1000 person-years (95% CI)	No. of births	No. of events	Rate per 1000 person-years (95% CI)	No. of births	No. of events	Rate per 1000 person-years (95% CI)
Spontaneous vaginal^a^	407 581	30 747	6.1 (6.1-6.2)	407 539	7123	1.4 (1.3-1.4)	407 519	1509	0.3 (0.3-0.3)
Vacuum	46 454	3868	6.7 (6.5-6.9)	46 461	995	1.7 (1.6-1.8)	46 457	189	0.3 (0.3-0.4)
Forceps	23 132	1749	6.2 (5.9-6.5)	23 129	515	1.8 (1.6-1.9)	23 129	74-78^b^	0.3-0.3 (0.2-0.3)^b^
Sequential instrument	3005	311	7.9 (7.1-8.8)	3008	64	1.5 (1.2-2.0)	3008	1-5^b^	0-0.1 (0-0.3)^b^
Second stage cesarean	23 929	1765	6.6 (6.3-6.9)	23 933	557	2.0 (1.9-2.2)	23 934	55	0.2 (0.1-0.3)

Abbreviations: ADHD, attention-deficit/hyperactivity disorder; ASD, autism spectrum disorder; ID, intellectual disability.

^a^ Spontaneous vaginal delivery data are reported only for descriptive purposes and are not intended for comparison with operative delivery.

^b^ The exact number of cases in the sequential instrument group and in the forceps group are suppressed and ranges are presented due to small cell counts to avoid back calculations.

**Figure 2.**
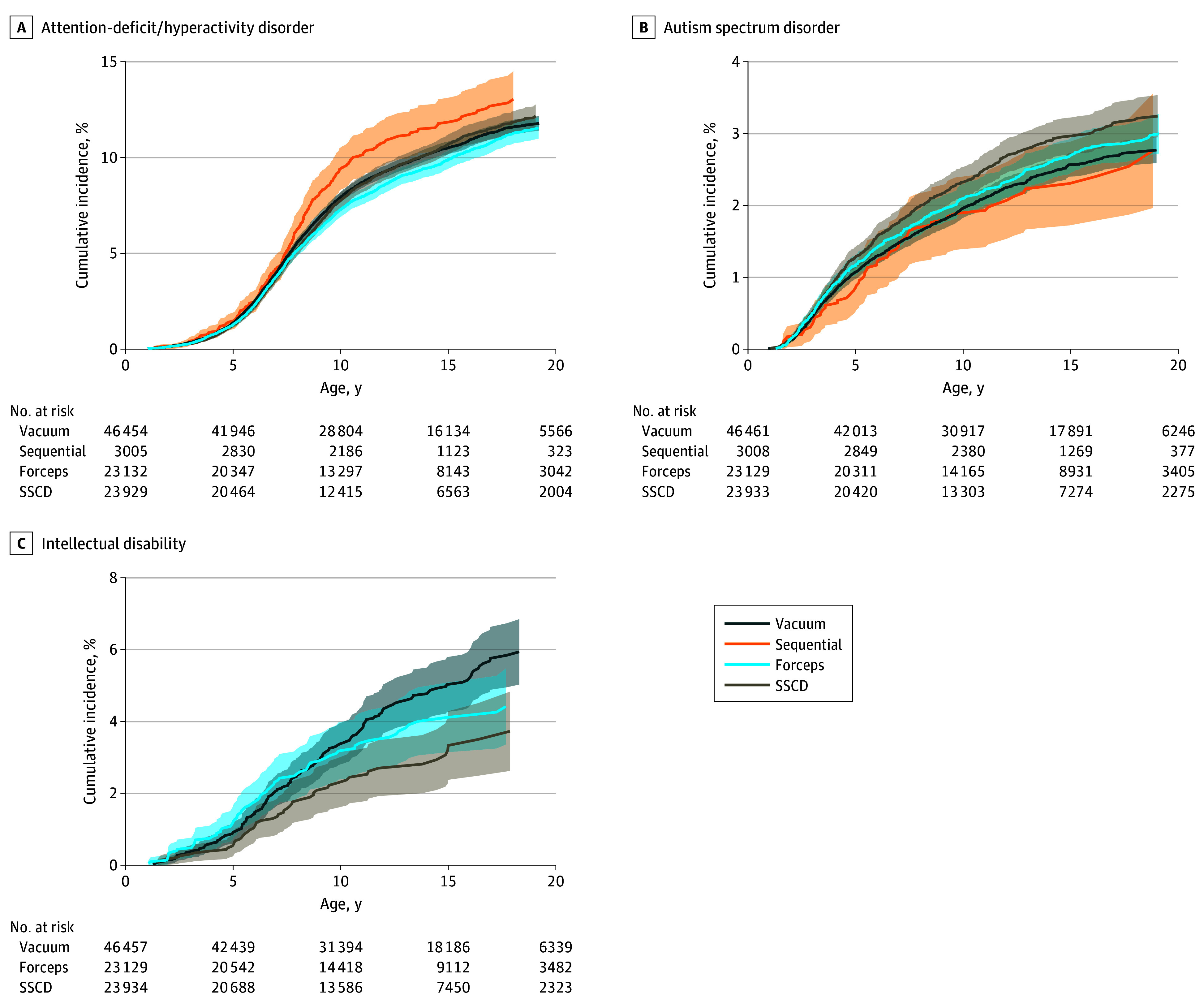
Cumulative Incidence of Neurodevelopmental Outcomes by Mode of Delivery in the Second Stage of Labor Estimates presented for attention-deficit/hyperactivity disorder (A), autism spectrum disorder (B), and intellectual disability (C) among children born at full term with no congenital anomalies in British Columbia, Canada, from 2000 to 2019. Shaded areas indicate 95% CIs. SSCD indicates second-stage cesarean delivery.

The rate of each neurodevelopmental outcome varied by mode of delivery. For instance, the highest ADHD rate was among children born after sequential instrument delivery (7.9 [95% CI, 7.1-8.8] per 1000 person-years), followed by vacuum delivery (6.7 [95% CI, 6.5-6.9] per 1000 person-years), SSCD (6.6 [95% CI, 6.3-6.9] per 1000 person-years), and forceps delivery (6.2 [95% CI, 5.9-6.5] per 1000 person-years) (Table 2). Patterns in ASD rates differed from ADHD, where all operative vaginal delivery groups had lower rates than SSCD (2.0 [95% CI, 1.9-2.2] per 1000 person-years). Rates of ID were comparable across modes of delivery, ranging from 0.2 (95% CI, 0.1-0.3) per 1000 person-years in the SSCD group to 0.3 (95% CI, 0.3-0.4) per 1000 person-years in the vacuum delivery group. Compared with SSCD, the probability of experiencing ADHD during follow-up was higher in the sequential instrument group and the probability of experiencing ID during follow-up was higher in the vacuum delivery group, while the probability of experiencing ASD was lower in the vacuum delivery group (Figure 2).

After adjustment, sequential instrument delivery was associated with a 13% higher rate of ADHD compared with SSCD (AHR, 1.13 [95% CI, 1.00-1.28]), while similar rates of ADHD were observed among forceps delivery, vacuum delivery, and SSCD (Table 3). Compared with SSCD, lower rates of ASD were associated with vacuum delivery, forceps delivery, and sequential instrument delivery; however, the results were not statistically significant. In addition, a 53% higher rate of ID was observed among vacuum deliveries compared with SSCD (AHR, 1.53 [95% CI, 1.12-2.10]).

**Table 3.** Crude and Adjusted Hazard Ratios for ADHD, ASD, and ID Among Full-Term, Nonanomalous Children Born During the Second Stage of Labor, British Columbia, Canada, 2000 to 2019^a^

Mode of delivery	Hazard ratio (95% CI)											
	ADHD (n = 96 520)				ASD (n = 96 531)				ID (n = 96 528)			
	Crude	*P* value	Adjusted	*P* value	Crude	*P* value	Adjusted	*P* value	Crude	*P* value	Adjusted	*P* value
Vacuum	1.00 (0.95-1.06)	.21	1.04 (0.98-1.10)	.97	0.85 (0.76-0.94)^b^	.002	0.95 (0.84-1.07)	.27	1.59 (1.18-2.14)^b^	.003	1.53 (1.12-2.10)^b^	.01
Forceps	0.95 (0.89-1.01)	.88	0.99 (0.93-1.06)	.11	0.91 (0.80-1.02)	.11	0.94 (0.84-1.05)	.40	1.31 (0.93-1.87)	.13	1.33 (0.93-1.91)	.11
Sequential instrument	1.14 (1.01-1.29)^b^	.04	1.13 (1.00-1.28)^b^	.03	0.79 (0.61-1.02)	.07	0.80 (0.62-1.04)	.10	0.48 (0.17-1.31)	.15	0.45 (0.16-1.24)	.12
Second-stage cesarean	1 [Reference]	NA	1 [Reference]	NA	1 [Reference]	NA	1 [Reference]	NA	1 [Reference]	NA	1 [Reference]	NA

Abbreviations: ADHD, attention-deficit/hyperactivity disorder; ASD, autism spectrum disorder; ID, intellectual disability; NA, not applicable.

^a^ Models adjusted for maternal age, parity, body mass index, smoking status, maternal history of neurodevelopmental or psychiatric disorders, preexisting and gestational diabetes, gestational hypertension, infant sex, birth weight of 4000 g or more, fetal distress, and fetal dystocia.

^b^ Statistically significant (*P* < .05).

Results were mostly unchanged in sensitivity analyses with missing data for BMI imputed and the removal of smoking status from our models (eTables 2 and 3 in Supplement 1). In both analyses, vacuum delivery remained associated with a heightened hazard of ID. When smoking status was removed, sequential instrument delivery was no longer associated with a higher hazard of ADHD (AHR, 1.12 [95% CI, 0.99-1.27]), although the overlapping 95% CIs between our main and sensitivity analyses suggests no meaningful difference in the results (eTable 3 in Supplement 1). Of the 288 303 births between 2002 and 2019, 15 377 (5.3%) were attempted by forceps and 31 182 (10.8%) were attempted by vacuum. The failure rate was 6.1% (937 of 15 377) among attempted forceps and 9.6% (2984 of 31 182) among attempted vacuum deliveries. Analyses categorizing attempted mode of delivery as successful or failed found failed vacuum delivery to confer a higher hazard of ADHD (AHR, 1.19 [95% CI, 1.04-1.35]) (eTable 4 in Supplement 1). Although main analyses found sequential instrument delivery to confer a higher hazard of ADHD and vacuum delivery to confer a higher hazard of ID, there were no associations between mode of delivery and each neurodevelopmental outcome when using an intention-to-treat framework.

## Discussion

In this cohort study of children born during the second stage of labor, we found that sequential instrument delivery was associated with an increased hazard of ADHD and vacuum delivery was associated with an increased hazard of ID when compared with SSCD. Statistically similar rates of ASD were found between modes of delivery. Overall, these findings suggest comparable safety of operative vaginal delivery and SSCD regarding neurodevelopmental outcomes among children.

Our study found differences in the incidence of ADHD by mode of delivery that partially mirrored, but also contrasted, prior literature. For instance, studies in Sweden found a higher incidence of ADHD in operative vaginal delivery compared with spontaneous vaginal delivery (9% vs 3%)^15^ and a 14% to 16% higher risk of ADHD among cesarean delivery.^16^ Descriptively, we found similar ADHD rates between spontaneous vaginal delivery and forceps delivery, with higher rates among vacuum delivery and SSCD. Studies conducted in other high-income countries have found no association between mode of delivery and ADHD when comparing forceps, vacuum, cesarean, and spontaneous vaginal deliveries.^25,27,28,29^ The departure of our results from those of the existing literature may be accounted for by differences in methodology, with previous studies using spontaneous vaginal delivery as a comparator group with operative vaginal delivery and SSCD as well as allocating forceps and vacuum deliveries into a larger operative vaginal delivery group. Despite the higher incidence of ADHD among sequential instrument delivery in our study, only 0.6% of children were born by this intervention, which had a minimal association with ADHD rates at the population level.

Studies have reported higher rates of ASD among cesarean deliveries compared with spontaneous vaginal delivery.^17,30^ However, Curran et al^13^ found that this association was accounted for by familial confounding, concluding that the elevated risk was attributable to shared genetic and/or environmental factors rather than the delivery mode itself. The similar incidence of ASD across mode of delivery groups in this study is consistent with prior literature, which suggests that delivery mode may not hold prognostic value for ASD.

We found that children who were born by vacuum delivery had a 53% higher risk of ID. However, there was a low baseline rate of ID (0.2 per 1000 person-years among SSCD) and a marginal absolute difference in rates between SSCD and vacuum delivery (0.1 per 1000 person-years). Thus, the clinical significance of this finding is likely minimal, suggesting comparable safety of vacuum delivery and SSCD. This finding is consistent with previous studies, which have found slightly lower mathematics test scores and grade point averages at age 16 years between children born after vacuum delivery compared with spontaneous vaginal delivery,^31^ similar rates of performance across the core domains of ID (ie, conceptual, social, and practicality) at 5 years of age among children born after an operative vaginal delivery compared with SSCD,^9,32^ and no difference in odds of ID between vacuum delivery and spontaneous vaginal delivery.^33^

### Strengths and Limitations

Our study has some strengths, including an analysis of a large, population-based cohort with over 20 years of follow-up. This allowed for a robust evaluation of the association between mode of delivery in the second stage of labor and neurodevelopmental outcomes in children, which has previously been hindered by small sample sizes.^9,15^ Through leveraging the richness of information within our data sources, we accounted for multiple confounders, such as smoking status, preexisting maternal conditions (ie, diabetes), and maternal psychiatric history. In addition, we included only second-stage deliveries that required an intervention, facilitating appropriate comparisons between operative vaginal delivery and SSCD.

Our study also has some limitations. It is constrained by the low sensitivity of the ASD diagnostic algorithm within British Columbia’s health administrative databases, resulting in an underrepresentation of ASD cases.^24^ However, this misclassification should not be differentially distributed by mode of delivery. In addition, relative estimates may have been biased by data quality concerns pertaining to BMI and smoking status. We mitigated these concerns through 2 sensitivity analyses and found similar results. Our results may be skewed due to survival bias, as children whose births had severe complications may have been more likely to die before the age of 1 year (start of follow-up). However, this bias is likely to be minimal given the small number of deaths within this study and the similar death rates between mode of delivery groups. Despite adjusting for multiple covariates, residual confounding may exist due to a lack of information on some confounders, such as sociodemographic (eg, race and ethnicity) and socioeconomic (eg, maternal income and educational level) characteristics. Moreover, we were unable to account for practitioner preference patterns. We did not consider a scenario where children received diagnoses of multiple neurodevelopmental disorders, limiting the generalizability of this work. Given the association between socioeconomic characteristics and ADHD, ASD, and ID and the comorbidity of these disorders,^34,35^ future work should consider these limitations.

## Conclusions

Through using the appropriate comparison groups, this cohort study demonstrated the comparable safety of mode of delivery interventions within the second stage of labor. Although previous research has raised concerns about the increased risk of neurodevelopmental disorders among operative vaginal and cesarean deliveries, our results add to a growing body of literature that suggest that these associations are likely confounded by the clinical indication for the intervention. To produce actionable and valid evidence, research on the optimal mode of delivery should compare people who present with similar clinical profiles rather than broad comparisons that do not account for the complexity of labor and delivery. By doing so, we can develop a better understanding of mode of delivery during the second stage of labor as a potentially modifiable risk factor for neurodevelopmental disorders in childhood.

## Notes

Supplement 1. **eMethods.** Description of Data Sources
eReferences
**eTable 1.** *International Classification of Disease, Ninth and Tenth Revision, Canada* (*ICD-9* and *ICD-10*), Case Definitions and Data Sources Used to Define Study Variables **eTable 2.** Crude and Adjusted Hazard Ratios (HRs) and 95% Confidence Intervals (CIs) for ADHD, ASD, and ID Using Multiple Imputation for Missing Data on Pre-Pregnancy Body Mass Index (Sensitivity Analysis), British Columbia, Canada, 2000-2019 **eTable 3.** Crude and Adjusted Hazard Ratios (HRs) and 95% Confidence Intervals (CIs) for ADHD, ASD, and ID Without Controlling for Smoking Status (Sensitivity Analysis), British Columbia, Canada, 2000-2019 **eTable 4.** Crude and Adjusted Hazard Ratios (HRs) and 95% Confidence Intervals (CIs) for ADHD, ASD, and ID Using an Intention-to-Treat Framework (Sensitivity Analysis), British Columbia, Canada, 2002-2019 **eFigure.** Schematic of Analysis Plan

Supplement 2.
Data Sharing Statement
