# Supplementary material for: Long-Term Neurodevelopmental Outcomes After Forceps, Vacuum, and Second-Stage Cesarean Delivery
Source: JAMA Netw Open. 2026 Jan 30;9(1):e2556637. doi: 10.1001/jamanetworkopen.2025.56637 (PMC12859721; doi:10.1001/jamanetworkopen.2025.56637)
Supplement: Supplement 1. — eMethods. Description of Data Sources eReferences eTable 1. International Classification of Disease, Ninth and Tenth Revision, Canada (ICD-9 and ICD-10), Case Definitions and Data Sources Used to Define Study Variables eTable 2. Crude and Adjusted Hazard Ratios (HRs) and 95% Confidence Intervals (CIs) for ADHD, ASD, and ID Using Multiple Imputation for Missing Data on Pre-Pregnancy Body Mass Index (Sensitivity Analysis), British Columbia, Canada, 2000-2019 eTable 3. Crude and Adjusted Hazard Ratios (HRs) and 95% Confidence Intervals (CIs) for ADHD, ASD, and ID Without Controlling for Smoking Status (Sensitivity Analysis), British Columbia, Canada, 2000-2019 eTable 4. Crude and Adjusted Hazard Ratios (HRs) and 95% Confidence Intervals (CIs) for ADHD, ASD, and ID Using an Intention-to-Treat Framework (Sensitivity Analysis), British Columbia, Canada, 2002-2019 eFigure. Schematic of Analysis Plan [file jamanetwopen-e2556637-s001.pdf]

Supplementary Online Content

Rajasingham M, Lisonkova S, Razaz N, Muraca GM. Long-term neurodevelopmental outcomes after forceps, vacuum, and second-stage cesarean delivery. *JAMA Netw Open.* 2026;9(1):e2556637. doi:10.1001/jamanetworkopen.2025.56637

eMethods. Description of Data Sources
eReferences
eTable 1. *International Classification of Disease, Ninth and Tenth Revision, Canada* (*ICD-9* and *ICD-10*), Case Definitions and Data Sources Used to Define Study Variables
eTable 2. Crude and Adjusted Hazard Ratios (HRs) and 95% Confidence Intervals (CIs) for ADHD, ASD, and ID Using Multiple Imputation for Missing Data on Pre-Pregnancy Body Mass Index (Sensitivity Analysis), British Columbia, Canada, 2000-2019
eTable 3. Crude and Adjusted Hazard Ratios (HRs) and 95% Confidence Intervals (CIs) for ADHD, ASD, and ID Without Controlling for Smoking Status (Sensitivity Analysis), British Columbia, Canada, 2000-2019
eTable 4. Crude and Adjusted Hazard Ratios (HRs) and 95% Confidence Intervals (CIs) for ADHD, ASD, and ID Using an Intention-to-Treat Framework (Sensitivity Analysis), British Columbia, Canada, 2002-2019
eFigure. Schematic of Analysis Plan

This supplementary material has been provided by the authors to give readers additional information about their work.

© 2026 Rajasingham M et al. *JAMA Network Open.*

eMethods. Description of Data Sources

A detailed description of each data sourced used in the study is described below:

Discharge Abstract Database, British Columbia
This database collects clinical, demographic, and administrative data from hospital discharges and day surgeries in British Columbia. Information is available from April 1, 1985, with diagnostic information ascertained from ICD-9 and 10 codes.[1]

Medical Services Plan, British Columbia
Canadian citizens and permanent residents can apply for publicly funded health insurance, which in British Columbia is called the Medical Service Plan (MSP). The MSP file contains fee-for-service outpatient claims as billed by specialists and primary care physicians. Data is available from April 1, 1985, with diagnostic information obtained from ICD-9 codes.[2]

PharmaNet
PharmaNet is a provincial network that monitors and records prescriptions dispensed by community pharmacies and some outpatient hospital pharmacies in British Columbia. Information is available from January 1, 1996, and includes Drug Identification Numbers which can be mapped on to the Anatomical Therapeutic Chemical (ATC) system.[3]

Vital Statistics Birth Database, British Columbia
The Vital Statistics Death database provides details on all births occurring in British Columbia since January 1, 1985.[4]

Vital Statistics Death Database, British Columbia
Vital Statistics Death database provides details on all deaths occurring in British Columbia since January 1, 1985.[5]

Central Demographics File
The Central Demographics File provides demographic (e.g., age, sex) and MSP registration information. Data is available from January 1, 1986.[6]

© 2026 Rajasingham M et al. *JAMA Network Open.*

eReferences

1. Canadian Institute for Health Information. Discharge Abstract Database (Hospital Separations) data set. In Population Data BC; 2022 [cited 2025 Feb 4]. Available from: https://www.popdata.bc.ca/data/health/dad

2. British Columbia Ministry of Health. Medical Services Plan data set. In Population Data BC; 2022 [cited 2025 Feb 4]. Available from: https://www.popdata.bc.ca/data/health/msp

3. British Columbia Ministry of Health. PharmaNet data set. In Population Data BC; [cited 2025 Feb 5]. Available from: https://www.popdata.bc.ca/data/health/pharmanet

4. British Columbia Vital Statistics Agency. BC Vital Events and Statistics Births data set. In Population Data BC; [cited 2025 July 25]. Available from: https://www.popdata.bc.ca/data/demographic/vs_births

5. British Columbia Vital Statistics Agency. British Columbia Vital Events and Statistics Deaths data set. In Population Data BC; [cited 2025 Feb 5]. Available from: https://www.popdata.bc.ca/data/demographic/vs_deaths

6. British Columbia Ministry of Health. Central Demographics File (MSP Registration and Premium Billings, Client Roster and Census Geodata)/Consolidation file (MSP registration and premium billing) data set. In Population Data BC; [cited 2025 Feb 5]. Available from: https://www.popdata.bc.ca/data/demographic/consolidation_file

© 2026 Rajasingham M et al. *JAMA Network Open.*

eTable 1. *International Classification of Disease, Ninth and Tenth Revision, Canada* (*ICD-9* and *ICD-10*), Case Definitions and Data Sources Used to Define Study Variables

	Variable coding	Case definition	Data sources
**Exclusions**			
Gestational age	Variable: GESTPERIOD<br><br>< 37 weeks or > 42 weeks		Vital Statistics Births
Pre-labour or first stage cesarean delivery	Variable: M_DUR_2MD_STAGE = missing or 0		BC Perinatal Data Registry
'Other' instrument delivery	Variable: M_MOD_DEL2 = 'other'		BC Perinatal Data Registry
Congenital anomaly	ICD-9: 740-759<br><br>ICD-10: Q00-Q99		Discharge Abstract Database, Medical Services Plan
Breech delivery	ICD-9: 652.11, 660.01<br><br>ICD-10: O32.101, O64.101 with diagnosis type 1, M, W, X, or Y		Discharge Abstract Database
**Exposure**			
Mode of delivery	Variable: M_MOD_DEL2<br><br>Forceps, vacuum, sequential instrument vs. second stage cesarean delivery		BC Perinatal Data Registry
Intention to treat delivery mode (sensitivity analysis)	ICD-10 and CCI:<br>Spontaneous vaginal delivery = 5.MD.50, 5.MD.51<br><br>Attempted forceps:<br>- Successful delivery: 5.MD.53<br>- Failed delivery: (5.MD.60.JZ, 5.MD.60.KC, 5.MD.60.RA, 5.MD.60.RE, 5.MD.60.JW, 5.MD.60.RG) and O66.5<br><br>Attempted vacuum:<br>- Successful delivery: 5.MD.54, 5.MD.55<br>- Failed delivery: (5.MD.60.RD, 5.MD.60.KA, 5.MD.60.KD, 5.MD.60.RB, 5.MD.60.RF, 5.MD.60.JX, 5.MD.60.RH, 5.MD.60.C) and O66.5<br><br>Cesarean delivery = 5.MD.60.KE, 5.MD.60.JY, 5.MD.60.KB, 5.MD.60.KG, 5.MD.60.KF, 5.MD.60.AA, 5.MD.60.KT<br><br>Attempted forceps, attempted vacuum vs. cesarean delivery<br><br>Failed vacuum, failed forceps, successful vacuum, successful forceps vs. cesarean delivery		Discharge Abstract Database

© 2026 Rajasingham M et al. *JAMA Network Open.*

	Variable coding	Case definition	Data sources
**Outcomes**			
ADHD	ICD-9: 314<br><br>ICD-10: F90<br><br>ACT: N06BA01 (amphetamine) N06BA02 (dexamfetamine), N06BA04 (methylphenidate), N06BA09 (atomoxetine), N06BA12 (lisdexamfetamine)	≥1H or ≥1P or ≥2RX	Discharge Abstract Database, Medical Services Plan, PharmaNet
ASD	ICD-9: 299<br><br>ICD-10: F84 (excluding F84.2)	≥1H or ≥2P	Discharge Abstract Database, Medical Services Plan
ID	ICD-9: 317-319<br><br>ICD-10: F70-73, F78-F79	≥1H or ≥1P	Discharge Abstract Database, Medical Services Plan
**Maternal covariates**			
Age	Variable: M_AGE<br><br>≤19, 20-24, 30-34, 35-39, ≥40 vs. 25-39		Vital Statistics Birth
Parity	Variable: PARITY<br><br>Nulliparous vs. parous		BC Perinatal Data Registry
Body mass index	Variable: M_BMI_NO<br><br>Underweight <18.5 kg/m2, overweight 25.0-29.9, obese ≥30 vs. normal 18.5-24.9		BC Perinatal Data Registry
Smoking status	Variable: SMOKER_TYPE_CD<br><br>Quit prior to pregnancy, continuous through pregnancy vs. none		BC Perinatal Data Registry
Mental health history (psychiatric and neurodevelopmental disorders)	ICD-9: 290-319<br><br>ICD-10: F00-F99		Discharge Abstract Database, Medical Services Plan
Pre-existing diabetes	Variable: R_IDDM (insulin dependent) and R_NIDDM (non-insulin dependent)		BC Perinatal Data Registry
Gestational diabetes	Variable: M_DIABETES		BC Perinatal Data Registry
Gestational hypertension	Variable: R_PIH		BC Perinatal Data Registry
**Fetal/infant covariates**			
Sex	Variable: C_SEX<br><br>Female vs. male		Vital Statistics Birth

© 2026 Rajasingham M et al. *JAMA Network Open.*

	Variable coding	Case definition	Data sources
High infant birthweight	Variable: B_ADMISSION_WEIGHT<br><br>≥ 4,000g vs. < 4,000g		BC Perinatal Data Registry
Fetal distress	ICD-9: 663.0, 663.1, 663.3, 663.4, 663.5<br><br>ICD-10: O68, O69		Discharge Abstract Database
Dystocia	ICD-9: 652, 653, 654.0-654.1, 654.4-654.9, 660.1-660.2, 660.4-660.9, 661.0-661.2, 661.4, 661.9, 662<br><br>ICD-10: O32, O33, O34.0, O34.1, O34.3-O34.9, O62.0-O62.2, O62.4, O62.8-O62.9, O63, O65, O66		Discharge Abstract Database

ACT, Anatomical Therapeutic Chemical; ADHD, attention deficit hyperactivity disorder; ASD, autism spectrum disorder; BC, British Columbia; ID, intellectual disability, H denotes a hospital admission, P denotes an outpatient visit, and Rx denotes a drug prescription.

© 2026 Rajasingham M et al. *JAMA Network Open.*

eTable 2. Crude and Adjusted Hazard Ratios (HRs) and 95% Confidence Intervals (CIs) for ADHD, ASD, and ID Using Multiple Imputation for Missing Data on Pre-Pregnancy Body Mass Index (Sensitivity Analysis), British Columbia, Canada, 2000-2019

	**ADHD (n = 96,520)**		**ASD (n = 96,531)**		**ID (n = 96,528)[a]**	
	Crude HR (95% CI)	aHR (95% CI)	Crude HR (95% CI)	aHR (95% CI)	Crude HR (95% CI)	aHR (95% CI)
Vacuum	1.00 (0.95, 1.06)	1.04 (0.98, 1.10)	**0.85 (0.76, 0.94)**	0.94 (0.83, 1.05)	**1.59 (1.17, 2.14)**	**1.54 (1.22, 1.86)**
Forceps	0.95 (0.89, 1.01)	1.00 (0.93, 1.07)	0.91 (0.80, 1.02)	0.95 (0.83, 1.07)	1.31 (0.93, 1.86)	1.34 (0.97, 1.69)
Sequential instrument	**1.14 (1.01, 1.29)**	**1.14 (1.02, 1.26)**	0.79 (0.61, 1.02)	0.81 (0.55, 1.07)	-	-
Second stage cesarean	REF	REF	REF	REF	REF	REF

ADHD, attention hyperactivity disorder; ASD, autism spectrum disorder; ID, intellectual disability; bold text indicates statistical significance (α < 0.05).
Model was adjusted for maternal age, parity, BMI, smoking status, maternal history of neurodevelopmental or psychiatric disorders, pre-existing and gestational diabetes, gestational hypertension, infant sex, high birth weight (≥ 4,000g), fetal distress, and dystocia.
[a]Sequential instrument was removed from the statistical models as we could not estimate adjusted hazard ratios due to small cell counts.

© 2026 Rajasingham M et al. *JAMA Network Open.*

eTable 3. Crude and Adjusted Hazard Ratios (HRs) and 95% Confidence Intervals (CIs) for ADHD, ASD, and ID Without Controlling for Smoking Status (Sensitivity Analysis), British Columbia, Canada, 2000-2019

	**ADHD (n = 96,520)**		**ASD (n = 96,531)**		**ID (n = 96,528)**	
	Crude HR (95% CI)	aHR (95% CI)	Crude HR (95% CI)	aHR (95% CI)	Crude HR (95% CI)	aHR (95% CI)
Vacuum	1.00 (0.95, 1.06)	1.04 (0.98, 1.10)	**0.85 (0.76, 0.94)**	0.94 (0.84, 1.04)	**1.59 (1.17, 2.14)**	**1.54 (1.12, 2.10)**
Forceps	0.95 (0.89, 1.01)	0.99 (0.92, 1.06)	0.91 (0.80, 1.02)	0.94 (0.84, 1.07)	1.31 (0.93, 1.86)	1.34 (0.93, 1.91)
Sequential instrument	**1.14 (1.01, 1.29)**	1.12 (0.99, 1.27)	0.79 (0.61, 1.02)	0.80 (0.62, 1.04)	0.48 (0.17, 1.31)	0.45 (0.16, 1.24)
Second stage cesarean	REF	REF	REF	REF	REF	REF

ADHD, attention hyperactivity disorder; ASD, autism spectrum disorder; ID, intellectual disability; bold text indicates statistical significance (α < 0.05).
Model was adjusted for maternal age, parity, BMI, maternal history of neurodevelopmental or psychiatric disorders, pre-existing and gestational diabetes, gestational hypertension, infant sex, high birth weight (≥ 4,000g), fetal distress, and dystocia.

© 2026 Rajasingham M et al. *JAMA Network Open.*

eTable 4. Crude and Adjusted Hazard Ratios (HRs) and 95% Confidence Intervals (CIs) for ADHD, ASD, and ID Using an Intention-to-Treat Framework (Sensitivity Analysis), British Columbia, Canada, 2002-2019

	**ADHD (n = 64,240)**			**ASD (n = 64,245)**			**ID (n = 64,248)**		
	Rate[a]	Crude HR (95%CI)	aHR (95%CI)	Rate	Crude HR (95%CI)	aHR (95%CI)	Rate	Crude HR (95%CI)	aHR (95%CI)
Mode of delivery by successful/failed instrument use									
Failed vacuum	8.2	**1.19 (1.05, 1.36)**	**1.19 (1.04, 1.35)**	1.9	0.87 (0.68, 1.13)	0.88 (0.68, 1.13)	-	-	-
Successful vacuum	6.6	0.99 (0.92, 1.06)	1.02 (0.95, 1.11)	1.9	**0.85 (0.75, 0.97)**	0.91 (0.80, 1.04)	0.3	**1.45 (1.00, 2.09)**	1.36 (0.93, 2.00)
Failed forceps	8.5	0.99 (0.77, 1.27)	1.04 (0.81, 1.33)	2.5	1.10 (0.73, 1.65)	1.10 (0.74, 1.66)	-	-	-
Successful forceps	6.1	0.88 (0.81, 0.97)	0.95 (0.87, 1.04)	2.1	0.94 (0.81, 1.09)	0.97 (0.83, 1.12)	0.3	1.21 (0.78, 1.88)	1.24 (0.79, 1.94)
Failed instrument[b]	-	-	-	-	-	-	0.1	0.47 (0.19, 1.19)	0.44 (0.17, 1.11)
Second stage cesarean	6.5	REF	REF	2.2	REF	REF	0.2	REF	REF
Mode of delivery by intention-to-treat									
Attempted vacuum	6.8	1.01 (0.94, 1.09)	1.05 (0.97, 1.13)	1.9	**0.85 (0.76, 0.97)**	0.97 (0.84, 1.13)	0.3	1.33 (0.92, 1.91)	1.22 (0.84, 1.79)
Attempted forceps	5.8	**0.89 (0.82 0.97)**	0.96 (0.88, 1.05)	2.1	0.95 (0.83, 1.10)	0.91 (0.80, 1.03)	0.3	1.19 (0.77, 1.83)	1.20 (0.84, 1.79)
Second stage cesarean	6.5	REF	REF	2.2	REF	REF	0.2	REF	REF

ADHD, attention hyperactivity disorder; ASD, autism spectrum disorder; ID, intellectual disability; bold text indicates statistical significance (α < 0.05). Model was adjusted for maternal age, parity, BMI, maternal history of neurodevelopmental or psychiatric disorders, pre-existing and gestational diabetes, gestational hypertension, infant sex, high birth weight (≥ 4,000g), fetal distress, and dystocia.

[a]Rates expressed per 1,000 person-years.

[b]Failed vacuum and failed forceps were collapsed into a ‘failed instrument’ group due to small cell counts (<5) in some groups.

© 2026 Rajasingham M et al. *JAMA Network Open.*

eFigure. Schematic of Analysis Plan

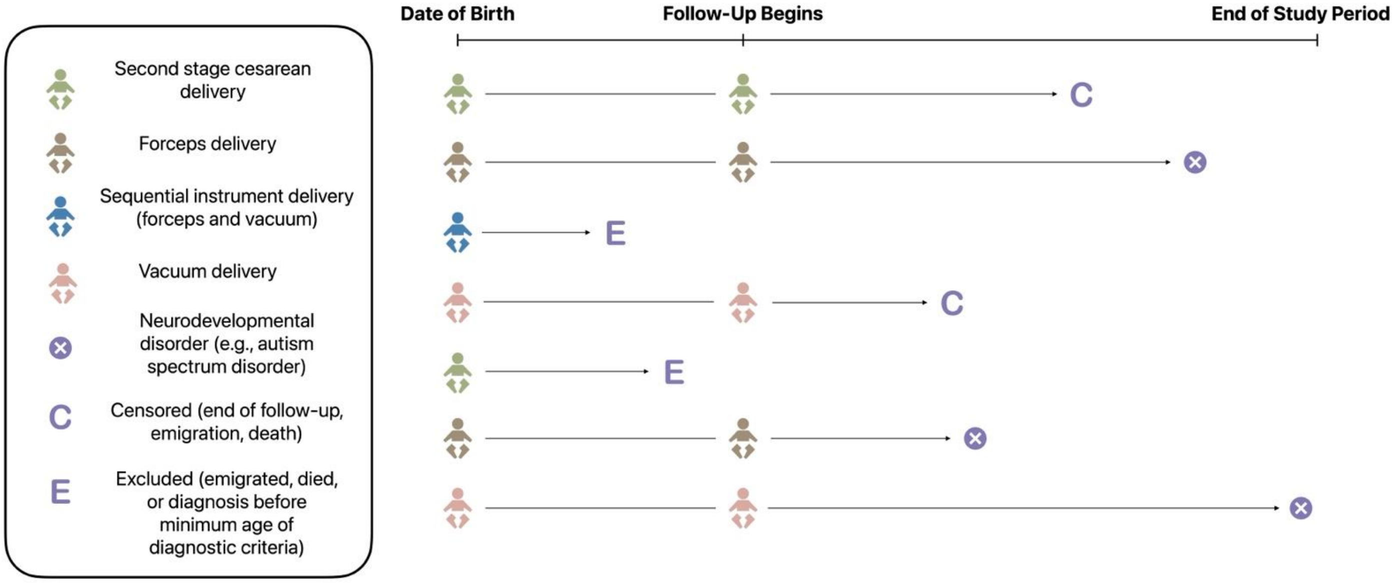

© 2026 Rajasingham M et al. *JAMA Network Open.*
