# Supplementary material for: Long-Term Neurodevelopmental Outcomes After Forceps, Vacuum, and Second-Stage Cesarean Delivery
Source: JAMA Netw Open. 2026 Jan 30;9(1):e2556637. doi: 10.1001/jamanetworkopen.2025.56637 (PMC12859721; doi:10.1001/jamanetworkopen.2025.56637)
Supplement: Supplement 2. — Data Sharing Statement [file jamanetwopen-e2556637-s002.pdf]

# Data Sharing Statement

Rajasingham. Long-Term Neurodevelopmental Outcomes After Forceps, Vacuum, and Second-Stage Cesarean Delivery. *JAMA Netw Open*. Published January 30, 2026. doi:10.1001/jamanetworkopen.2025.56637

**Data**
**Data available:** No

**Additional Information**
**Explanation for why data not available:** Access to data used in this study is provided by the Data Stewards at Population Data BC and is subject to approval but can be requested for research projects through the Data Stewards or their designated service providers. The following data sets were used in this study: Discharge Abstract Database (DAD), Medical Service Plan (MSP), Perinatal Data Registry (PDR), PharmaNet, Vital Statistics Births, Vital Statistics Deaths, Central Demographics File. You can find further information regarding these data sets by visiting the PopData project webpage at: https://my.popdata.bc.ca/project_listings/20-178/collection_approval_dates. All inferences, opinions, and conclusions drawn in this publication are those of the author(s) and do not reflect the opinions or policies of the Data Steward(s).
